# Modelled impact of a multi-cancer early detection screening programme on the demand for diagnostics in England

**DOI:** 10.1038/s41416-025-03331-8

**Published:** 2026-01-31

**Authors:** Joanne Martin, David A. Jones, Libby Ellis, Ewan Gray, Katharine Halliday, Sara Hiom, Sean McPhail, Andrew Millar, Willie Hamilton

**Affiliations:** 1 Queen Mary University of London, London, UK; 2 GRAIL Bio UK Ltd., London, UK; 3 Nottingham University Hospitals NHS Trust, Nottingham, UK; 4 National Disease Registration Service, NHS England, London, UK; 5 Royal Free London NHS Foundation Trust, London, UK; 6 University of Exeter, Exeter, UK

**Keywords:** Cancer screening, Cancer epidemiology

## Abstract

**Background:**

People with a cancer signal detected via multi-cancer early detection (MCED) screening need timely access to confirmatory diagnostic testing. We estimated the likely change in demand for diagnostic testing in England if MCED screening were introduced.

**Methods:**

Diagnostic demand was modelled based on (1) estimates of the volume of people aged 50–79 years who would be referred for diagnostic investigation following a ‘cancer signal detected’ result after MCED screening and (2) MCED test performance metrics. Predicted usage was compared with current annual usage using routine NHS datasets.

**Results:**

In an established MCED screening programme, assuming 70% of the total eligible population is screened annually (~13 million), the relative change in diagnostic demand was greatest for colonoscopy (+2.09%; +13,730 each year); the greatest absolute change was for computed tomography (CT; +0.76%; +62,320). This equates to +1040 colonoscopies and +4720 CT scans for every million screened.

**Conclusions:**

The predicted relative increase in diagnostic testing generated by MCED screening is small, though a large eligible population and maximum uptake could translate into a large number of procedures. Cancer diagnoses brought forward in time through screening should reduce diagnostic use for symptomatic presentations in the future.

## Background

Multi-cancer early detection (MCED) tests screen simultaneously for two or more cancer types using a biological specimen, e.g., blood, to detect cancer signals before symptomatic presentation and diagnosis in usual care [1–3].

The clinical utility of one blood-based MCED test (Galleri^®^; GRAIL, Inc., Menlo Park, CA, USA) for population screening in asymptomatic individuals aged 50–79 years in England is being assessed in the large, randomised controlled NHS-Galleri trial (NCT05611632) [1]. This MCED test analyses methylation patterns on cell-free DNA. Detection of a methylation pattern associated with cancer is returned as a cancer signal detected (CSD) test result (‘positive’ test result); when a cancer signal is detected, further analysis predicts the tissue type or organ associated with the cancer signal (cancer signal origin; CSO). The CSO is not a cancer diagnosis but rather can be used to guide diagnostic investigation to confirm if cancer is present.

Modelling has predicted that MCED screening could achieve substantial reductions in late-stage (stage III and IV) cancer incidence and cancer mortality if it were offered alongside current screening programmes in England [4]. To realise these benefits, people for whom a cancer signal is detected need timely, appropriate diagnostic investigation and subsequent treatment and care.

The initial diagnostic test choice in the NHS depends on the suspected cancer type and anatomical site, and may include biopsy, endoscopy, and/or imaging [5]. Estimating the potential impact of MCED screening on different diagnostic modalities is important for future workforce and capacity planning; however, little is currently known about how a novel MCED screening programme might impact diagnostic demand.

In this study, we modelled how a putative future Galleri MCED screening programme could impact demand for different diagnostic modalities in England, including imaging, biopsy, and endoscopy. For possible future scenarios, where more cancers are detected asymptomatically through multi-cancer screening, this study and its partner study examining the impact on cancer treatment [6] could support workforce planning and service delivery.

## Methods

We used a decision tree model to predict diagnostic use by modality, with the following inputs: (1) estimates of the volume of people who would be referred for diagnostic investigation due to a CSD test result if an annual MCED screening programme were introduced in England, based on performance data for the Galleri MCED test [7]; and (2) estimates of the distribution of CSOs in the screened population, based on the inferred distribution and accuracy of the predicted CSO reported by the MCED test.

Diagnostic use was estimated for two scenarios: (1) an initial (prevalent) screening round following the addition of MCED screening to current screening programmes, representing a population aged between 50 and 79 years attending their first screen; and (2) an established (steady-state) annual screening programme, representing a population in which individuals enter an MCED screening programme at 50 years of age and participate in annual MCED screening until 79 years of age. In this scenario the number of cancers found via MCED screening will have reached an equilibrium.

Our model did not account for any potential reductions in future diagnostic use that should occur with MCED screening due to cancer diagnoses being brought forward in time, or any changes in population size or cancer incidence. It also made no assumptions about the impact of demographic characteristics, such as socioeconomic status or ethnicity, on screening uptake or diagnostic use.

### Input data

We used three inputs to estimate the volume of people who would be referred for diagnostic investigation if an annual MCED screening programme were added to current screening programmes. The first was the total eligible screening population, taken from Office for National Statistics (ONS) mid-year population estimates for people aged 50–79 years in England for 2022 [8]. The second input was the estimated level of screening participation (50% to reflect a low participation scenario, 70% to reflect approximate participation in current screening programmes in the NHS in England [9], and 100% to reflect the maximum theoretical impact of MCED screening), which assumed no sociodemographic variation in screening participation; we also calculated the annual volume of people referred for diagnostic investigation for every 1 million screened as a scalable metric to demonstrate the impact of a phased roll-out. The third was the predicted proportion of CSD test results when using the MCED test for asymptomatic screening (1.4%; hereafter referred to as the ‘CSD rate’) [7].

The primary diagnostic modality used to investigate any given CSD result was determined by the predicted CSO. We could not estimate the distribution of predicted CSOs in the intended use population directly from existing clinical studies due to differences in population demographics [7] or study design [10]. Instead, a previously-published natural history (‘interception’) model [4, 11] was used to predict the distribution of cancer types that would be detected by MCED screening (i.e. ‘true positive’ test results). The cancer types were subsequently mapped back to the most applicable CSO to provide the estimated distribution of true positive CSO calls. The distribution of CSOs for apparent ‘false positive’ test results (defined in this study as those for which there was a CSD result but with no cancer diagnosed within 12 months) was assumed to be the same as for true positive results (i.e. the distribution of cancer types from the natural history model). The number of apparent false positive results was calculated from the number of true positive results and the assumed positive predictive value of the test (PPV; 43.1% [7]).

To identify the primary diagnostic investigation(s) likely to be undertaken for each CSD result, we mapped each of 18 possible CSOs to standard-of-care diagnostic procedures in England. To do this, we used clinical guidance developed for the NHS-Galleri trial to support the referral of trial participants with a CSD result directly into secondary care for diagnostic testing, and expert clinical input (Table S1). The version of the Galleri test modelled here (18 possible CSOs, 1 reported) is an optimised version of the test used in the NHS-Galleri trial (21 possible CSOs, 2 reported).

The diagnostic modalities included in our study and the CSOs for which they were modelled were: biopsy (cervix, haematopoietic and lymphoid, skin), colonoscopy (colon and rectal), computed tomography (CT; kidney, liver/bile duct, lung, pancreatic, gallbladder), cystoscopy (bladder, urothelial tract), flexible sigmoidoscopy (anal), gastroscopy (stomach, oesophageal), magnetic resonance imaging (MRI; bone and soft tissue, prostate), and ultrasound (breast, head and neck, ovarian, thyroid, uterus). These categories are necessarily broad to match NHS diagnostic activity statistics [12], but it should be noted that they are not exhaustive. We did not include any imaging procedures used for staging purposes, because these would have occurred anyway in the diagnostic workup of cancers presenting symptomatically. In the primary analysis, we did not include biopsies that were additional to the primary diagnostic modality, such as those obtained during endoscopy or after an initial ultrasound or CT. However, it is reasonable to assume that most cancers detected by MCED screening would have a biopsy as part of the diagnostic investigation. In a sensitivity analysis, we included biopsy for all true positive CSD results, in addition to the primary diagnostic modality.

### Model

**Fig. 1:**
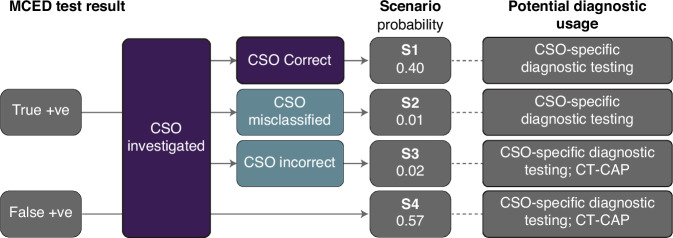
Decision tree model showing potential diagnostic resource use following cancer signal detected multi-cancer early detection (MCED) test results. ‘CSO misclassified’ means that the predicted CSO was incorrect, but the diagnostic test led to detection of a different cancer type. Probabilities are correct to two decimal places. CSO cancer signal origin, CT-CAP computed tomography of the chest, abdomen and pelvis.

We developed a decision tree model to map possible outcomes from a CSD test result, enabling the subsequent estimation of diagnostic resource use (Fig. 1). In brief, the decision tree first distributes CSD test results between true and false positives based on the positive predictive value of the MCED test (43.1%) [7]. For true positive test results, there were three possible scenarios: the predicted CSO was correct (‘CSO correct’); the predicted CSO was incorrect but the primary CSO-specific diagnostic test led to the detection of a different cancer type (‘CSO misclassified’); or the CSO was incorrect and cancer was not found with the primary diagnostic test, but with a subsequent computed tomography of the chest, abdomen and pelvis (CT-CAP; ‘CSO incorrect’). True positives for which the CSO was correct or misclassified were modelled to receive one primary diagnostic test; false positives and true positives for which the CSO was incorrect were modelled to receive two diagnostic tests (the CSO-specific diagnostic test followed by a CT-CAP). The distribution of true positive CSD MCED test results across these scenarios was based on a common percentage accuracy across all CSOs (93% [13]; data on file), and an assumed probability of 25% that diagnostic testing for incorrect CSOs led to the detection of a different cancer type, based on MCED test performance in a symptomatic population [14].

### Sensitivity analyses

We performed a one-way sensitivity analysis to assess the impact of changing the model input parameters using a range of plausible values, informed by published literature. For the CSD rate (which had a base assumption of 1.4% [7] for an average-risk population aged 50–79 years), alternative values of 0.9% [15] (the most conservative estimate for an asymptomatic screening population) and 1.5% (in a high-risk population) [7] were assessed. For CSO accuracy (which had a base assumption of 93%), a more conservative value of 88% was assessed [16]. For the probability that the CSO was incorrect but the primary diagnostic test led to the detection of a different cancer type (which had a base assumption of 25%), alternative values of 10% and 40% were assessed. For the PPV (which had a base assumption of 43.1%), alternative values of 31% (lower 95% CI) and 56% (Upper 95% CI) were assessed, though emerging real-world data [17, 18] suggest a higher PPV than previously published.

### Outputs

We modelled the change in use of the primary diagnostic modalities likely to be offered with each CSO based on standard clinical practice. Where diagnostic resolution was not reached after primary investigation of the predicted CSO (scenarios 3 and 4 in Fig. 1), we assumed that CT-CAP was carried out.

To estimate the difference in diagnostic use with MCED screening for all modalities except biopsy, we compared modelled outcomes with total annual activity data from the NHS Monthly Diagnostic Waiting Times and Activity dataset for 2023–24 [12].

We used a different approach to estimate the difference in biopsy use with MCED screening, because biopsy data are not included in the NHS Monthly Diagnostic Waiting Times and Activity dataset. As a comparator for this modality, we multiplied the estimated proportion of all registered cancers in England in 2019 that were microscopically verified (88.9% [19]) by the total number of cancer registrations in the same year (528,600 [19]) to estimate the number of biopsies performed in the diagnosis of cancer. We used 2019 data because they were the most recently available when this study was conducted, and were less likely to be affected by the COVID-19 pandemic. Instances in which biopsy was performed for suspected cancers, but with no cancer subsequently diagnosed, or for other conditions, were not captured in this comparator. Thus, this comparator represented an estimate of the number of biopsies specifically for diagnosed cancers, including benign brain tumours, in situ neoplasms, neoplasms of uncertain or unknown behaviour, and non-melanoma skin cancer. This differs from the Monthly Waiting Times and Activity data used as a comparator for the other diagnostic modalities included in our study, which capture all use regardless of indication (i.e. including non-cancer uses).

## Results

### Diagnostic activity in England following the initial introduction of a potential future MCED screening programme

For every 1 million people screened (at a 1.4% CSD rate), an estimated 14,000 CSD results would be referred for diagnostic investigation.

Table 1 shows the modelled change in diagnostic activity following the initial introduction of MCED screening in England, compared with current annual activity in England. For every one million individuals screened, the greatest relative increases were in colonoscopy (+0.49%; +3180 absolute increase) and gastroscopy (+0.28%; +2010), compared with NHS diagnostic activity in 2023–24. There was also a noteworthy relative increase for every million screened in biopsy activity (+0.32%; +1520) compared with all cancer biopsy activity in 2019. The greatest absolute increase for every million screened was in CT (+0.16%; +13,180). The relative change in diagnostic activity specifically attributable to false positives for these four modalities was 0.28% for colonoscopy (+1820), 0.16% for gastroscopy (+1140), 0.18% for biopsy (+860), and 0.13% for CT (+10,760) (Table S2). The estimated change in diagnostic activity attributable to true or false positives is driven by the PPV; a higher PPV (such as 56%, as modelled in a sensitivity analysis) would lower the estimates attributable to false positives.

**Table 1:** Predicted Change in Annual Diagnostic Activity Following the Initial Introduction of an MCED Screening Programme for individuals aged 50–79 years in England under different scenarios: for every 1 million screened, and full roll-out to eligible population with 50%, 70% and 100% participation.

Diagnostic modality	Total activity (*n*) in England (2023-24)^a^	For every 1 million screened		50% participation^b^ (~9.4 million screened)		70% participation^b^ (~13.2 million screened)		100% participation^b^ (~18.9 million screened)	
		Annual activity (*n*) associated with MCED screening^c^	Percentage change (%) in total annual activity^d^	Annual activity (*n*) associated with MCED screening^c^	Percentage change (%) in total annual activity^d^	Annual activity (*n*) associated with MCED screening^c^	Percentage change (%) in total annual activity^d^	Annual activity (*n*) associated with MCED screening^c^	Percentage change (%) in total annual activity^d^
Biopsy^e^	470,000	1520	0.32	14,370	3.06	20,120	4.28	28,740	6.12
Colonoscopy	656,100	3180	0.49	30,030	4.58	42,050	6.41	60,070	9.16
CT	8,221,600	13,180	0.16	124,400	1.51	174,100	2.12	248,700	3.03
Cystoscopy	344,600	300	0.09	2840	0.82	3970	1.15	5680	1.65
Flexible sigmoidoscopy	200,900	87	0.04	820	0.41	1140	0.57	1640	0.81
Gastroscopy	723,200	2010	0.28	18,950	2.62	26,530	3.67	37,910	5.24
MRI	4,346,300	380	0.01	3580	0.08	5010	0.12	7150	0.17
Ultrasound	8,267,800	2880	0.04	27,160	0.33	38,020	0.46	54,310	0.66

*CT* computed tomography, *MRI* magnetic resonance imaging.

^a^Data presented to the nearest hundred.

^b^The total population eligible for MCED screening was based on 2022 mid-population estimates by 5-year age band from 50 to 79 years of age (50%: 9,434,433 individuals; 70%: 13,208,206 individuals; 100%: 18,868,865 eligible individuals) [7].

^c^Data presented to the nearest ten (or nearest unit when <100).

^d^Data presented to two decimal places.

^e^Biopsy total activity data were from 2019 and include only biopsies for diagnosed cancer.

At 50% MCED screening participation (i.e. assuming 50% of the entire eligible population are screened in one year, which equates to approximately 9.4 million people screened, and 131,600 being ‘followed up’), the greatest relative increases in activity were for colonoscopy (+4.58%; +30,030) and biopsy (+3.06%; +14,370), and the greatest absolute increase was for CT (+1.51%; +124,400). At 100% participation (the maximum impact scenario, screening 18.9 million people in one year), these values doubled (+9.16%, +60,070 for colonoscopy; +6.12%, +28,740 for biopsy; +3.03%, +248,700 for CT).

### Diagnostic activity in England in a steady-state potential future MCED screening programme

Table 2 shows the modelled change in diagnostic activity in a steady-state MCED screening programme in England, for every one million individuals screened and for 50%, 70% and 100% participation scenarios. A steady-state programme represents a scenario in which individuals enter annual MCED screening aged 50 years, and participate until they are 79 years of age; it thus includes those participating in their first year of screening, and those participating in subsequent years.

**Table 2:** Predicted change in annual diagnostic activity in a steady-state multi-cancer early detection (MCED) screening programme for individuals aged 50–79 years in England under different scenarios: for every 1 million screened, and full roll-out to eligible population with 50%, 70% and 100% participation.

Diagnostic modality	Total activity (n) in England (2023-24)^a^	For every 1 million screened		50% participation^b^ (~9.4 million screened)		70% participation^b^ (~13.2 million screened)		100% participation^b^ (~18.9 million screened)	
		Annual activity (*n*) associated with MCED screening^c^	Percentage change (%) in total annual activity^d^	Annual activity (*n*) associated with MCED screening^c^	Percentage change (%) in total annual activity^d^	Annual activity (*n*) associated with MCED screening^c^	Percentage change (%) in total annual activity^d^	Annual activity (*n*) associated with MCED screening^c^	Percentage change (%) in total annual activity^d^
Biopsy^e^	470,000	560	0.12	5280	1.12	7390	1.57	10,550	2.25
Colonoscopy	656,100	1040	0.16	9800	1.49	13,730	2.09	19,610	2.99
CT	8,221,600	4720	0.06	44,510	0.54	62,320	0.76	89,020	1.08
Cystoscopy	344,600	99	0.03	930	0.27	1300	0.38	1860	0.54
Flexible sigmoidoscopy	200,900	35	0.02	330	0.17	470	0.23	670	0.33
Gastroscopy	723,200	700	0.10	6630	0.92	9280	1.28	13,260	1.83
MRI	4,346,300	180	<0.01	1730	0.04	2430	0.06	3470	0.08
Ultrasound	8,267,800	1070	0.01	10,080	0.12	14,110	0.17	20,160	0.24

*CT* computed tomography, *MRI* magnetic resonance imaging.

^a^Data presented to the nearest hundred.

^b^The total population eligible for MCED screening was based on 2022 mid-population estimates by 5-year age band from 50 to 79 years of age (50%: 9,434,433 individuals; 70%: 13,208,206 individuals; 100%: 18,868,865 eligible individuals) [7].

^c^Data presented to the nearest ten (or nearest unit when <100).

^d^Data presented to two decimal places.

^e^Biopsy total activity data were from 2019 and include only biopsies for diagnosed cancer.

The greatest relative increases for every one million screened in a steady-state screening programme were in colonoscopy (+0.16%; +1040) and gastroscopy (+0.10%; +700; Table 2). This is the same pattern as was estimated following the initial introduction of the MCED screening programme (Table 1), but the magnitude of change compared with current annual activity in England is smaller. There was also a noteworthy relative increase for every million screened in biopsy activity (0.12%; +560). The greatest absolute increase for every million screened was in CT use (+0.06%; +4720). The relative change in diagnostic activity attributable to false positives for these four modalities was 0.09% for colonoscopy (+590), 0.06% for gastroscopy (+400), 0.07% for biopsy (+320), and 0.05% for CT (+3860) (Table S3).

For the different participation scenarios, the pattern of change in diagnostic activity in a steady-state screening programme (Table 2) is likewise the same as following the initial introduction of MCED screening (Table 1), again with a smaller change. At 50% MCED screening participation in the total eligible population, the greatest relative increases in activity were for colonoscopy (+1.49%; +9800), and biopsy (+1.12%; +5280), and the greatest absolute increase was for CT (+0.54%; +44,510). At 100% participation, these values roughly doubled (+2.99%, +19,610 for colonoscopy; +2.25%, +10,550 for biopsy; +1.08%, +89,020 for CT).

### Sensitivity analysis

#### Test performance

Our sensitivity analysis demonstrated that our estimates are likely to be most sensitive to changes in the CSD rate, but less so to changes in CSO accuracy or the probability of a CSO misclassification. This pattern is illustrated for CT scans in Fig. S1, assuming 70% screening participation rate (results for all modalities at 70% screening participation are shown in Table S4). Modelling a higher PPV, and hence reducing the number of apparent false positives requiring an additional diagnostic test (CT-CAP), reduced the estimated number of CTs but had little impact on estimates for other diagnostic modalities (Fig. S1 and Table S4).

#### Biopsy

Including biopsy for all true positive CSD results, in addition to the primary diagnostic modality, would mean a substantially higher increase in activity: +1.47% (+6900) for every million screened in the initial screening round and 0.53% (+2490) in a steady-state screening programme, compared with all cancer biopsy activity in 2019 (Table S5).

## Discussion

We estimate that there is likely to be a small relative initial increase in demand for diagnostic testing if MCED screening were offered alongside current cancer screening programmes in the future. With a very large screening-eligible population and maximum theoretical uptake, a small percentage increase for each diagnostic modality could translate into a substantial increase in the absolute number of diagnostic procedures in the short term. Future reductions in diagnostic use from symptomatic presentations avoided due to detection by screening should moderate this impact in the longer term.

Activity is increasing year-on-year for most diagnostic modalities in our study under current standard-of-care practices in England [20–22]; as such, the estimated increase in diagnostic activity associated with MCED screening is small by comparison. For example, the maximum possible annual increases in demand for colonoscopy and gastroscopy in a steady-state scenario were predicted to be 2.99% and 1.83%, respectively, if all 18.9 million eligible people participated in MCED screening each year. This is in comparison to the estimated 6–7.5% annual increase in the number of endoscopy procedures between 2017 and 2019 [23]. The predicted annual 1% increase in demand for CT in the same maximum scenario is in comparison to a 6.8% increase in the annual number of CT scans between 2014/15 and 2018/19 [20]. The larger predicted increase in diagnostic demand after the initial introduction of a MCED screening programme (compared with the steady-state programme) is an expected short term impact driven partly by the underlying prevalence of undiagnosed cancer in the screening-eligible population. Endoscopy services in particular are under strain [20, 23, 24] and may come under further pressure from changes to current screening programmes, such as lowering the referral threshold for the faecal immunochemical test (FIT) [25]. Improvements to screening outlined in the NHS Long Term Plan [26], such as the rollout of targeted lung cancer screening, will also increase diagnostic activity, particularly the use of CT scans, in the short term [27]. However, as with MCED screening, the introduction of other screening approaches should reduce diagnostic activity related to symptomatic presentation in the longer term, as cancers are increasingly detected asymptomatically via screening.

The increase in diagnostic activity associated with MCED screening is predicted to persist, albeit to a lesser extent, in a long-term steady-state screening programme. If MCED screening were introduced in England in the future, more cancer types, including less common cancers for which single-cancer screening programmes are unlikely, would have the opportunity to be detected earlier, before symptoms appear. Once a screening programme has reached a steady state, the continued additional diagnostic activity will be largely driven by false positive test results. Even with a high PPV, some diagnostic activity, related to the investigation of people who are not diagnosed with cancer following diagnostic workup, will remain. Interestingly, recent real-world data on MCED test use in the US has shown that 9% of individuals with a CSD test result from an initial MCED test, but a negative initial confirmatory diagnostic test, went on to be diagnosed with cancer following a second MCED test within 10 months [28].

The benefits of MCED tests that predict a CSO (tissue type or organ) to streamline the diagnostic process have recently been explored. One modelling study predicted that a diagnostic workup based on a given CSO could result in a more efficient process than a non-directed workup, but also that continued investigation for cancers in locations other than the CSO is justifiable [29]. Both of these scenarios are incorporated into our model. A CSO-directed diagnostic process is also likely to be similar to the investigation of a patient presenting with site-specific symptoms of suspected cancer, and as such may be more easily incorporated into current clinical practice.

Our study focused on the initial diagnostic test(s) to confirm or rule out cancer following MCED screening. Our model did not include procedures related directly to the staging of cancers once detected, nor did it include activity to support treatment decisions, such as planning for surgery or radiotherapy. However, it is acknowledged that patients who are diagnosed with cancer (regardless of the route to diagnosis, whether through screening or symptomatic presentation) are likely to have a biopsy and/or imaging, in addition to the initial diagnostic tests. In our study, biopsy was the primary diagnostic investigation for cervical, skin, lymphoid and haemopoetic cancers, but adding biopsy as a secondary modality for all cancers detected by MCED screening would increase demand for histopathology even further in the short term. Assuming no overdiagnosis, all cancers detected by MCED screening would present symptomatically in the future, so with the exception of some activity for false positive CSDs, these biopsies are not additional in the longer term. We expect almost all to require biopsy regardless of route to diagnosis. Cancers diagnosed by initial ultrasound or CT may also require a second interventional radiology procedure.

The magnitude of change in activity estimated in our study varied by diagnostic modality, with the greatest proportional increases in activity for colonoscopy, gastroscopy, and biopsy. The modelled increases in colonoscopy and gastroscopy are due in part to the greater prevalence of and relatively high sensitivity of the MCED test for gastrointestinal cancers [10]. The comparator for the estimated increase in demand for biopsies with MCED screening was biopsies related to diagnosed cancer, whereas the comparator for other modalities was all activity irrespective of indication. Therefore, the comparative increase in biopsies (as a primary diagnostic investigation) with MCED screening is likely to be an overestimate compared with other modalities.

Diagnostic investigations for people who receive a CSD result may have other impacts on health services. The diagnostic process may coincidentally identify precursor or pre-invasive lesions (unrelated to the CSD result) or incidental findings that require further investigation [30]. Whilst adding to diagnostic activity, the discovery of precancerous lesions has potential benefits for both individuals and health services. For example, identification and removal of colorectal polyps can prevent bowel cancer, thus reducing requirements for cancer diagnosis and treatment in future. The potential impact of precancerous lesions and incidental findings on the health service, such as the requirement for surveillance, and their optimal management is not yet known; however, results from the NHS-Galleri trial will provide crucial insights on this. A small number of patients might also incur harm from diagnostic investigations [31] after MCED screening, especially if they undergo two or more diagnostic procedures. However, the overall impact of this is likely to be relatively small.

In the event of a CSD result for which the recommended diagnostic tests are carried out, but no cancer is found, the point at which clinicians should cease diagnostic investigation and return individuals to usual care has yet to be established [32]; this will also impact diagnostic activity and clinical workloads. Some clinicians may therefore offer repeat CTs for surveillance in patients in whom no cancer is found after CSD result. This could mean that our predictions for CT use are underestimated. Previous research showed that 31% of individuals with a CSD result on both an initial and a repeat MCED test are diagnosed with cancer within six months, while none of those with a ‘no CSD’ (i.e. negative) result on repeat test were diagnosed with cancer in the subsequent 16.7 months [28]. In practice, it may be more efficient to repeat an MCED test, as has become routine practice with the Galleri test in the US.

Careful planning to manage the capacity of diagnostic services, especially endoscopy, histopathology and imaging, would be required prior to introduction of any MCED screening programme in the future. Phased rollout of MCED screening, either geographically or by age, could provide the opportunity to test the assumptions made in this modelling (e.g., multiple diagnostic tests for false positives) and validate the predicted additional diagnostic activity. The NHS, government, and cancer and health charities have proposed specific approaches aiming to increase and upskill the diagnostic workforce, increase and upgrade diagnostic equipment, and re-organise diagnostic services to meet increasing demand [20, 33, 34]. The results of our modelling study could support workforce and resource planning for implementation of MCED screening and other screening innovations in the NHS.

We did not explicitly model any interplay between MCED screening and current cancer screening programmes, or make any assumptions about impact on uptake. Whilst MCED screening is not intended to be a replacement, there is likely to be some degree of overlap, in addition to the detection of cancers in individuals who fall outside the age- or risk-based eligibility criteria. It is not yet possible to quantify any potential overlap, nevertheless, the impact on diagnostic demand is likely to be minimal if similar diagnostic modalities are used to investigate ‘positive’ results.

### Strengths and limitations

This study applied an evidence-based model to routine NHS data, using a range of input parameters from previous clinical and modelling studies. Input was sought from a wide range of clinicians to ensure the diagnostic approaches in the model reflected current practice for diagnosing suspected cancer and to validate the model results.

This model could be updated in future with sensitivity estimates for new or improved MCED tests, and revised assumptions based on evidence from MCED use in real-world settings and in large, randomised, controlled clinical trials such as the NHS-Galleri trial.

Some carefully considered decisions and assumptions were made in order for our model to function, given the lack of data on MCED screening in England. However, people with CSD test results may be investigated differently in a real-world clinical scenario.

Our study focused on the initial diagnostic test conducted following a CSD test result, accounting for the greater number of tests required when a cancer signal is detected but no cancer is subsequently diagnosed [10]. In practice, some individuals for whom a cancer signal is detected and cancer is subsequently diagnosed, may undergo more than one diagnostic test to achieve resolution, which may increase diagnostic activity more than is estimated here. Similarly, some early stage cancers may also be more difficult to diagnose and require a greater number of diagnostic procedures to achieve resolution than late stage cancers. In addition, our model assumed that people for whom no cancer is found during the first diagnostic procedure (i.e. for an incorrect CSO prediction or a false positive result) would undergo a CT-CAP as a second diagnostic procedure, after which no further investigations would be undertaken. However, more than two diagnostic procedures may be carried out in some of these cases.

The type of diagnostic procedure used for people with a CSD MCED test result could also be different in real-world clinical practice than in our model. Our estimates are based on how diagnostic tests are used to investigate cancer in symptomatic people under current standard-of-care in the NHS, but the diagnostic tests used to investigate asymptomatic people referred from an MCED screening programme may differ. We modelled change in activity for broad categories of diagnostic modalities, which was necessary in order to use NHS diagnostic activity statistics; our study, therefore, may miss some nuanced effects of MCED screening by modality. Other tests, such as FIT for colorectal cancer, may also support diagnostic decision making following the detection of a cancer signal with an MCED test; however, it is not yet clear how other diagnostic or triage tests might fit into diagnostic pathways for MCED screening.

MCED screening will ‘re-route’ some cancers from symptomatic diagnostic pathways (including emergency presentations, urgent suspected cancer referrals and GP referrals) [35], due to more cancers being diagnosed before symptoms occur. However, in the steady-state screening programme scenario, we did not directly account for the fact that annual MCED screening would result in some cancers being diagnosed in earlier years, thus reducing diagnostic activity from symptomatic presentation in subsequent years. In practice, the overall impact of MCED screening on diagnostic demand (at steady-state) will be a combination of reduced activity from symptomatic presentation and some increases in activity associated with apparent false positive results and repeat testing.

The CSD rate and PPV used to estimate the number of cancers found by the MCED test in this study implicitly account for any overdiagnosis, as these values come from real-world, prospective cohort studies. However, the interception model used to estimate the proportion of each CSO detected by the MCED test assumes no overdiagnosis. This is due to the low likelihood of overdiagnosis considering the test’s preferential detection of more aggressive (cfDNA-shedding) cancers. However, these CSO proportions could differ if the MCED test led to overdiagnosis in certain cancers.

The screening-eligible population size is based on adults aged 50–79 years in England in 2022. We do not define a specific timepoint at which a MCED screening programme might be rolled out and reach steady-state. The population of England is projected to increase and to age: between 2022 and 2032, the screening-eligible population is projected to increase by approximately 5%, from 18.9 million to 19.9 million [36]. With a growing and aging population, the number of cancer cases is projected to rise. A small increase in age-standardised cancer incidence rates (~1%) is also predicted over a similar timeframe [37]. Our model does not attempt to build in an inflationary increase in the number of people undergoing diagnostic testing following a CSD result at a future time point, but an increase in absolute numbers is expected.

Finally, participation rates in this study did not account for known sociodemographic differences in screening participation. For example, participation in breast and bowel cancer screening in England is lower among people in more deprived socioeconomic groups [38–40]. Little is currently known about how sociodemographic factors might affect participation in MCED screening compared with current screening programmes, but any differences could be minimised if known barriers to participation are addressed.

## Conclusions

Offering MCED screening alongside current screening programmes in England is estimated to generate a relatively small increase in demand for diagnostic testing. Assuming current standard-of-care diagnostic tests, the greatest increases in diagnostic activity estimated to be associated with MCED screening were for colonoscopy and gastroscopy.

This study contributes to a small but growing evidence base that will support diagnostic workforce and resource planning for the potential addition of MCED screening to current screening programmes in future. Our model can be updated as new data become available on MCED test sensitivity, cancer type distribution, and other model assumptions; thus, estimates of the impact of MCED screening on diagnostic resource use can be refined over time.

## Supplementary information

Supplemental Material

## Data Availability

Data will be made available upon reasonable request to the corresponding author.

## Code availability

The code used to generate the results in this study are available on request from the corresponding author.
