# Supplementary material for: Modelled impact of a multi-cancer early detection screening programme on the demand for diagnostics in England
Source: Br J Cancer. 2026 Jan 31;134(8):1190–7. doi: 10.1038/s41416-025-03331-8 (PMC13035848; doi:10.1038/s41416-025-03331-8)
Supplement: Supplementary file 1 — Supplemental Material [file 41416_2025_3331_MOESM1_ESM.docx]

**Supplementary Materials**

**
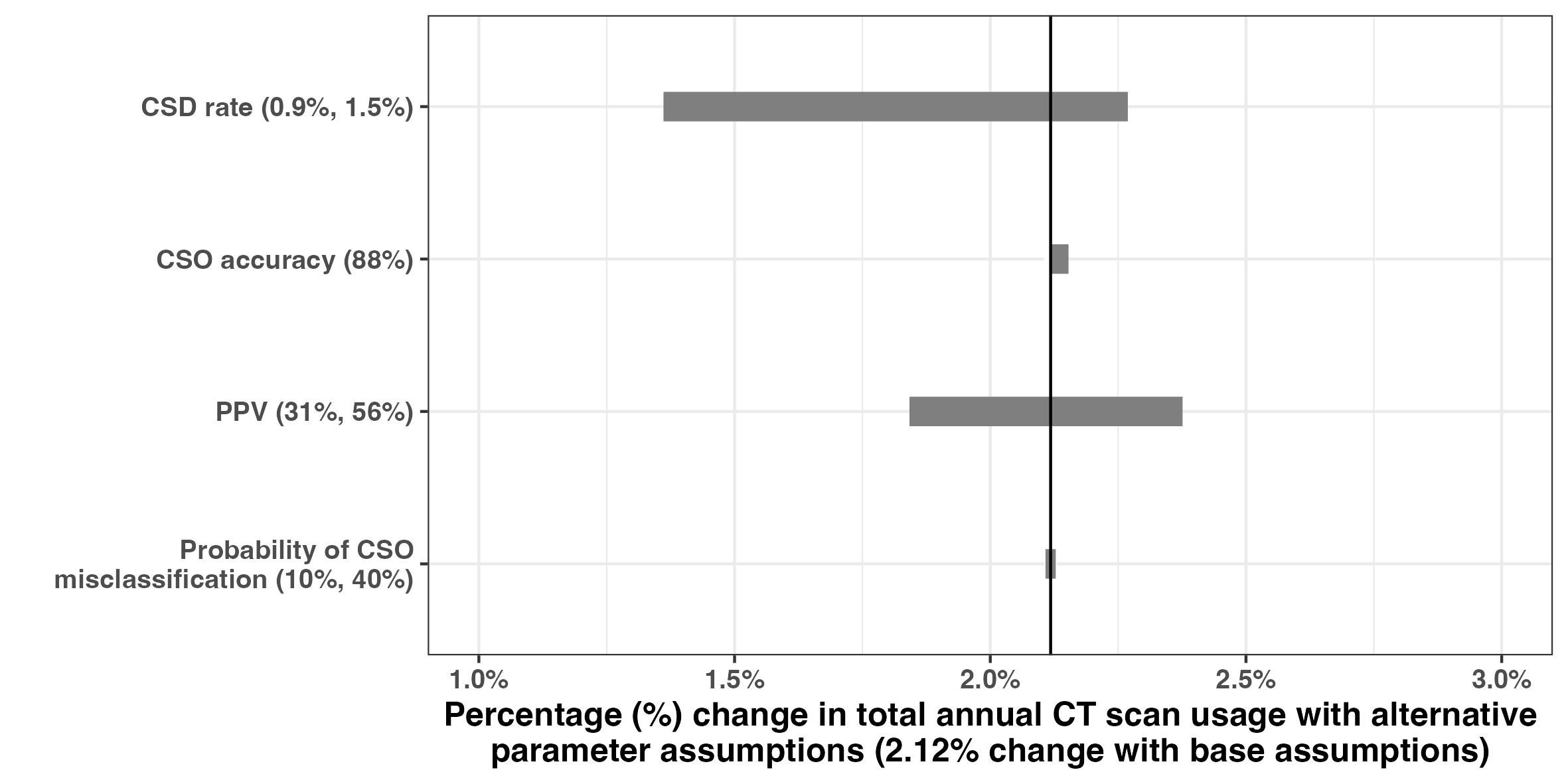
**

**Figure S1. Percentage Change in Total Annual Computed Tomography (CT) Scan Usage For Various Alternative Model Input Parameters Compared With Change Reported With Base Assumptions.** The model input parameters modified were the cancer signal detected (CSD) rate (base assumption: 1.4%), cancer signal origin (CSO) accuracy (base assumption: 93%), positive predictive value (PPV; base assumption: 43.1%), and probability of CSO misclassification (base assumption: 25%). Results are shown for a 70% participation rate only, but patterns are the same across all screening participation rates and for every million screened.

**Table S1. Multi-Cancer Early Detection (MCED) Test Cancer Signal Origins (CSOs) Mapped To Diagnostic Procedures.**

| **Cancer signal origin** | **Standard of care diagnostic procedure(s)** | **Primary investigation used in model^a,b^**  (mapped to NHS Diagnostics Waiting Times and Activity [DM01]) |
| --- | --- | --- |
| Anus | Flexible Sigmoidoscopy/ Colonoscopy, Physical exam, Proctoscopy, Biopsy | Flexible Sigmoidoscopy |
| Bladder, Urothelial Tract | Flexible Cystoscopy, CT Abdomen and Pelvis, Urine Cytology | Flexible Cystoscopy |
| Bone and Soft Tissue | MRI with Contrast/CT Chest, Abdomen and Pelvis, X-ray, Biopsy | MRI |
| Breast^a^ | Physical Examination, Mammography/Ultrasound, Biopsy | Ultrasound |
| Cervix | Speculum Exam +/- Biopsy +/- Cervical Screening (HPV Test +/- Cytology if overdue), MRI Pelvis, Colposcopy, Biopsy | Biopsy |
| Colon, Rectum | Colonoscopy/CT Colonography, Colon Capsule Endoscopy, Biopsy | Colonoscopy |
| Haematopoietic and Lymphoid Organs | Blood Test (FBC, peripheral blood film, biochemistry screen, LDH, viral serology [Hep B, C, HIV], Beta 2 microglobulin, immunoglobulins [including protein electrophoresis, serum free light chains, urine protein electrophoresis]), Biopsy (various, including bone marrow biopsy) | Biopsy |
| Head and Neck^b^ | Endoscopy, MRI/CT/Ultrasound, Laryngoscopy, Fibreoptic Nasal Endoscopy, Contrast CT, Biopsy | Gastroscopy, Ultrasound |
| Kidney | Urine Test for Blood, CT Renal (pre- and post- contrast), Biopsy | CT |
| Liver, Bile Duct | Blood Test (tumour markers), CT (triple phase of the liver to include pancreas) | CT |
| Lung | CT Thorax, X-ray, Biopsy | CT |
| Melanocyte-Containing Tissue/Skin | Skin Inspection, Panendoscopy of Oropharynx (if appropriate), CT Chest, Abdomen and Pelvis, Biopsy | Biopsy |
| Ovary | CA125 Blood Test, Transvaginal Ultrasound Scan/MRI Pelvis, CT | Ultrasound |
| Pancreas, Gallbladder | Blood Test (complete/comprehensive blood panels including CA19.9, HbA1c), Dual Phase Contrast-Enhanced CT | CT |
| Prostate | Blood Test (PSA level), Digital Rectal Examination, Multi-Parametric MRI of the Prostate, Targeted Biopsy | MRI |
| Stomach, Oesophagus | Upper Gastrointestinal Endoscopy, CT, Cytosponge, Biopsy | Gastroscopy |
| Thyroid | Ultrasound Imaging of the Thyroid, Biopsy, Cytology | Ultrasound |
| Uterus | Transvaginal Ultrasound/MRI Pelvis, Pipelle Biopsy | Ultrasound |

Standard of care investigation would also likely include a blood test for all suspected cancer types, with the possible exception of bone. CA125: cancer antigen 125; CA19.9: carbohydrate antigen 19.9; CT: computed tomography; FBC: full blood count; HbA1c: haemoglobin A1C; Hep: hepatitis; HIV: human immunodeficiency virus; HPV: human papillomavirus; LDH: lactate dehydrogenase; MRI: magnetic resonance imaging; PET: positron emission tomography; PSA: prostate specific antigen.

^a^DM01 statistics do not include mammography, so this was not included as the primary diagnostic procedure for a breast CSO

^b^One primary investigation was included in the model for all CSOs except head and neck, which has two; this is due to the diverse anatomical locations included in this CSO.

**Table S2. Predicted Change in Annual Diagnostic Activity For Every One Million Screened Following the Initial Introduction of a Multi-Cancer Early Detection (MCED) Screening Programme for individuals aged 50–79 years in England: estimated annual activity attributable to false positives**

| **Diagnostic modality** | **Total activity (n) in England (2023-24)^a^** | **Estimated annual activity (n) associated with MCED screening^b^** | **Percentage change (%) in total annual activity** | **Estimated annual activity (n) attributable to false positives^b^** | **Percentage change (%) in activity attributable to false positives** |
| --- | --- | --- | --- | --- | --- |
| **Biopsy^c^** | **470,000** | **1520** | **0.32** | **860** | **0.18** |
| **Colonoscopy** | **656,100** | **3180** | **0.49** | **1820** | **0.28** |
| **CT** | **8,221,600** | **13,180** | **0.16** | **10,760** | **0.13** |
| **Cystoscopy** | **344,600** | **300** | **0.09** | **170** | **0.05** |
| **Flexible sigmoidoscopy** | **200,900** | **87** | **0.04** | **49** | **0.02** |
| **Gastroscopy** | **723,200** | **2010** | **0.28** | **1140** | **0.16** |
| **MRI** | **4,346,300** | **380** | **0.01** | **210** | **0.01** |
| **Ultrasound** | **8,267,800** | **2880** | **0.04** | **1630** | **0.02** |

^a^Data presented to the nearest hundred. ^b^Data presented to the nearest ten (or nearest unit when <100). ^c^Biopsy data were from 2019 and include only biopsies for diagnosed cancer.

CT (Computed Tomography), MRI (Magnetic Resonance Imaging)

**Table S3. Predicted Change in Annual Diagnostic Activity For Every One Million Screened in a Steady-State Multi-Cancer Early Detection (MCED) Screening Programme for individuals aged 50–79 years in England: estimated annual activity attributable to false positives**

| **Diagnostic modality** | **Total activity (n) in England (2023-24)^a^** | **Estimated annual activity (n) associated with MCED screening^b^** | **Percentage change (%) in total annual activity** | **Estimated annual activity (n) attributable to false positives^b^** | **Percentage change (%) in activity attributable to false positives** |
| --- | --- | --- | --- | --- | --- |
| **Biopsy^c^** | **470,000** | **560** | **0.12** | **320** | **0.07** |
| **Colonoscopy** | **656,100** | **1040** | **0.16** | **590** | **0.09** |
| **CT** | **8,221,600** | **4720** | **0.06** | **3860** | **0.05** |
| **Cystoscopy** | **344,600** | **99** | **0.03** | **56** | **0.02** |
| **Flexible sigmoidoscopy** | **200,900** | **35** | **0.02** | **20** | **0.01** |
| **Gastroscopy** | **723,200** | **700** | **0.10** | **400** | **0.06** |
| **MRI** | **4,346,300** | **180** | **<0.01** | **100** | **<0.01** |
| **Ultrasound** | **8,267,800** | **1070** | **0.01** | **610** | **0.01** |

^a^Data presented to the nearest hundred. ^b^Data presented to the nearest ten (or nearest unit when <100). ^c^Biopsy data were from 2019 and include only biopsies for diagnosed cancer.

CT (Computed Tomography), MRI (Magnetic Resonance Imaging)

**Table S4. Sensitivity Analysis To Assess The Impact Of Changing The Model Input Parameters.** The table shows the percentage (%) change in usage for each diagnostic modality for the set of model input parameters used in the analysis in this manuscript (‘base assumptions’), then the percentage change with one aspect of the parameters changed (as listed in the header row of the table). The model input parameters modified were the cancer signal detected (CSD) rate (base assumption: 1.4%), probability of cancer signal origin (CSO) misclassification (base assumption: 25%), CSO accuracy (base assumption: 93%), and positive predictive value (PPV; base assumption: 43.1%). Results are shown for a 70% participation rate only, but patterns are the same across all screening participation rates and for every million screened.

| **Initial screening round** | | | | | | | | |
| --- | --- | --- | --- | --- | --- | --- | --- | --- |
| **Diagnostic modality** | **Base assumptions** | **0.9% CSD rate** | **1.5% CSD rate** | **10% CSO misclassified** | **40% CSO misclassified** | **88% CSO accuracy** | **31% PPV** | **56% PPV** |
| **Biopsy** | 4.28% | 2.75% | 4.59% | 4.28% | 4.28% | 4.29% | 4.28% | 4.28% |
| **Colonoscopy** | 6.41% | 4.12% | 6.87% | 6.41% | 6.41% | 6.39% | 6.42% | 6.40% |
| **CT** | 2.12% | 1.36% | 2.27% | 2.13% | 2.11% | 2.14% | 2.38% | 1.84% |
| **Cystoscopy** | 1.15% | 0.74% | 1.24% | 1.15% | 1.15% | 1.16% | 1.15% | 1.15% |
| **Flexible sigmoidoscopy** | 0.57% | 0.37% | 0.61% | 0.57% | 0.57% | 0.57% | 0.57% | 0.57% |
| **Gastroscopy** | 3.67% | 2.36% | 3.93% | 3.67% | 3.67% | 3.68% | 3.67% | 3.67% |
| **MRI** | 0.12% | 0.07% | 0.12% | 0.12% | 0.12% | 0.12% | 0.12% | 0.12% |
| **Ultrasound** | 0.46% | 0.30% | 0.49% | 0.46% | 0.46% | 0.46% | 0.46% | 0.46% |
| **Steady-state screening programme** | | | | | | | | |
| **Diagnostic modality** | **Base assumptions** | **0.9% CSD rate** | **1.5% CSD rate** | **10% CSO misclassified** | **40% CSO misclassified** | **88% CSO accuracy** | **31% PPV** | **56% PPV** |
| **Biopsy** | 1.57% | 1.01% | 1.68% | 1.57% | 1.57% | 1.57% | 1.57% | 1.57% |
| **Colonoscopy** | 2.09% | 1.34% | 2.24% | 2.09% | 2.09% | 2.09% | 2.09% | 2.09% |
| **CT** | 0.76% | 0.49% | 0.81% | 0.76% | 0.75% | 0.76% | 0.85% | 0.66% |
| **Cystoscopy** | 0.38% | 0.24% | 0.41% | 0.38% | 0.38% | 0.38% | 0.38% | 0.38% |
| **Flexible sigmoidoscopy** | 0.23% | 0.15% | 0.25% | 0.23% | 0.23% | 0.23% | 0.23% | 0.23% |
| **Gastroscopy** | 1.28% | 0.82% | 1.38% | 1.28% | 1.28% | 1.29% | 1.28% | 1.28% |
| **MRI** | 0.06% | 0.04% | 0.06% | 0.06% | 0.06% | 0.06% | 0.06% | 0.06% |
| **Ultrasound** | 0.17% | 0.11% | 0.18% | 0.17% | 0.17% | 0.17% | 0.17% | 0.17% |

**Table S5. Sensitivity Analysis To Assess The Impact Of Adding Biopsy For All True Positive CSD results**

The table shows the predicted change in annual number of biopsies associated with MCED screening if all true positive CSD results (all CSOs) had a biopsy as part of the diagnostic investigation, for every 1 million screened, and full roll-out to the eligible population with 50%, 70% and 100% participation.

| **Diagnostic modality** | **Total activity (n) in England (2023-24)^a^** | **For every 1 million screened** | | **50% participation^b^**  **(~9.4 million screened)** | | **70% participation^b^**  **(~13.2 million screened)** | | **100% participation^b^**  **(~18.9 million screened)** | |
| --- | --- | --- | --- | --- | --- | --- | --- | --- | --- |
|  |  | **Annual activity (n) associated with MCED screening^c^** | **Percentage change (%) in total annual activity^d^** | **Annual activity (n) associated with MCED screening^c^** | **Percentage change (%) in total annual activity^d^** | **Annual activity (n) associated with MCED screening^c^** | **Percentage change (%) in total annual activity^d^** | **Annual activity (n) associated with MCED screening^c^** | **Percentage change (%) in total annual activity^d^** |
|  |  |  |  |  |  |  |  |  |  |
| **Initial screening round** | | | | | | | | | |
| **Biopsy^e^** | 470,000 | 6900 | 1.47 | 65,080 | 13.85 | 91,120 | 19.39 | 130,200 | 27.69 |
| **Steady-state screening programme** | | | | | | | | | |
| **Biopsy^e^** | 470,000 | 2490 | 0.53 | 23,470 | 4.99 | 32,860 | 6.99 | 46,940 | 9.99 |

^a^Data presented to the nearest hundred. ^b^The total population eligible for MCED screening was based on 2022 mid-population estimates by 5-year age band from 50–79 years of age (50%: 9,434,433 individuals; 70%: 13,208,206 individuals; 100%: 18,868,865 eligible individuals) [7]. ^c^Data presented to the nearest ten (or nearest unit when <100). ^d^Data presented to two decimal places. ^e^Biopsy total activity data were from 2019 and include only biopsies for diagnosed cancer.

CT (Computed Tomography), MRI (Magnetic Resonance Imaging)
